# HIV prevalence among men who have sex with men in Brazil: results of the 2nd national survey using respondent-driven sampling

**DOI:** 10.1097/MD.0000000000010573

**Published:** 2018-05-25

**Authors:** Ligia Kerr, Carl Kendall, Mark Drew Crosland Guimarães, Rosa Salani Mota, Maria Amélia Veras, Inês Dourado, Ana Maria de Brito, Edgar Merchan-Hamann, Alexandre Kerr Pontes, Andréa Fachel Leal, Daniela Knauth, Ana Rita Coimbra Motta Castro, Raimunda Hermelinda Maia Macena, Luana Nepomuceno Costa Lima, Lisangela Cristina Oliveira, Maria do Socorro Cavalcantee, Adele Schwartz Benzaken, Gerson Pereira, Cristina Pimenta, Ana Roberta Pati Pascom, Ximena Pamela Diaz Bermudez, Regina Célia Moreira, Luis Fernando Macedo Brígido, Ana Cláudia Camillo, Willi McFarland, Lisa G. Johnston

**Affiliations:** a Faculdade de Medicina, Universidade Federal do Ceará, Departamento de Saúde Comunitária, Fortaleza, CE, Brasil; b Department of Global Community Health and Behavioral Sciences, Tulane University, New Orleans, LA, USA, Faculdade de Medicina da Universidade Federal do Ceará, Saúde Comunitária, Fortaleza, CE; c Departamento de Medicina Preventiva e Social, Universidade Federal de Minas Gerais, Belo Horizonte, MG; d Departamento de Estatística e Matemática Aplicada, Universidade Federal do Ceará, Fortaleza, CE; e Departamento de Saúde Coletiva, Faculdade de Ciências Medicas da Santa Casa de São Paulo, São Paulo, SP; f Instituto de Saúde Coletiva, Universidade Federal da Bahia, Salvador, BA; g Centro de Pesquisas Aggeu Magalhaes, Recife, PE; h Centro de Ciências da Saúde – Departamento de Saúde Coletiva, Universidade de Brasília Faculdade de Ciências da Saúde, Brasília; i Instituto de Psicologia, Universidade Federal do Rio de Janeiro, Rio de Janeiro; j Departamento de Sociologia; k Departamento de Medicina Social, Universidade Federal do Rio Grande do Sul, Porto Alegre, RS; l FIOCRUZ – Unidade Mato Grosso do Sul, Universidade Federal do Mato Grosso do Sul, Campo Grande, MS; m Faculdade de Medicina da Universidade Federal do Ceará, Faculdade de Medicina – Departamento de Fisioterapia, Fortaleza, CE; n Instituto Evandro Chagas, Ananindeua, PA; o Centro Universitário Autônomo do Brasil, Curitiba, PR; p Secretaria de Saúde do Estado do Ceará, Secretaria de Saúde do Município de Fortaleza, Fortaleza, CE; q Departamento de IST e HIV/AIDS e Hepatites Virais, Ministério da Saúde, Brasília; r Centro de Ciências da Saúde, Universidade de Brasília, Brasília, DF; s Instituto Adolfo Lutz, São Paulo – SP; t Fundação Alfredo da Matta, Manaus, AZ, Brasil; u Center for Global Health, University of California, San Francisco, CA; v Tulane School of Public Health and Tropical Medicine, Department of Global Community Health and Behavioral Sciences, New Orleans, LA, USA.

**Keywords:** Brazil, HIV prevalence, MSM, respondent-driven sampling

## Abstract

This paper reports human immuno-deficiency virus (HIV) prevalence in the 2nd National Biological and Behavioral Surveillance Survey (BBSS) among men who have sex with men (MSM) in 12 cities in Brazil using respondent-driven sampling (RDS).

Following formative research, RDS was applied in 12 cities in the 5 macroregions of Brazil between June and December 2016 to recruit MSM for BBSS. The target sample size was 350 per city. Five to 6 seeds were initially selected to initiate recruitment and coupons and interviews were managed online. On-site rapid testing was used for HIV screening, and confirmed by a 2nd test. Participants were weighted using Gile estimator. Data from all 12 cities were merged and analyzed with Stata 14.0 complex survey data analysis tools in which each city was treated as its own strata. Missing data for those who did not test were imputed HIV+ if they reported testing positive before and were taking antiretroviral therapy.

A total of 4176 men were recruited in the 12 cities. The average time to completion was 10.2 weeks. The longest chain length varied from 8 to 21 waves. The sample size was achieved in all but 2 cities.

A total of 3958 of the 4176 respondents agreed to test for HIV (90.2%). For results without imputation, 17.5% (95%CI: 14.7–20.7) of our sample was HIV positive. With imputation, 18.4% (95%CI: 15.4–21.7) were seropositive.

HIV prevalence increased beyond expectations from the results of the 2009 survey (12.1%; 95%CI: 10.0–14.5) to 18.4%; CI95%: 15.4 to 21.7 in 2016. This increase accompanies Brazil's focus on the treatment to prevention strategy, and a decrease in support for community-based organizations and community prevention programs.

## 1 Introduction

Although global evidence shows an overall reduction in Acquired Immune Deficiency Syndrome (AIDS) cases in many countries,^[1,2]^ the human immuno-deficiency virus (HIV) epidemic among men who have sex with men (MSM) in low-, middle-, and high-income countries appears to be expanding.^[3–7]^ MSM are at high risk for HIV infection because of the syndemic of structural, biological, and behavioral vulnerabilities that act together to increase the chances of being infected.^[3–5,8,9]^

In Latin America, between 2000 and 2015, the number of new HIV infections among adults has slowly risen. From 2010 to 2015, Brazil, a country with a history of an exemplary AIDS prevention program, is now among those countries in Latin America (LA) and Caribbean where HIV infection among adults has increased (UNAIDS 2016). Brazil has the largest population in the region and accounts for more than 41% of the total new infections occurring among 7 countries: Argentina, the Bolivarian Republic of Venezuela, Colombia, Cuba, Guatemala, Mexico, and Peru.^[1]^

Brazil presents great socioeconomic inequalities, and the AIDS epidemic mirrors this inequality. From 2006 to 2015, AIDS rates in the more developed South and Southeast regions of Brazil demonstrated a reduction of 7.4% and 23.4%, respectively. However, Porto Alegre, a state capital in the South region, reported a very high rate of 74 AIDS cases/100,000 inhabitants during the same period, twice the rate of the rest of the state and 4 times the Brazilian average. On the other hand, the North and Northeast, the poorest regions in the country, showed a linear increase in AIDS over the same period. AIDS rates increased from 14.9 to 24.0 cases/100,000 inhabitants in North region, and 11.2 to 15.3 cases/100,000 inhabitants in the Northeast, representing a growth of 61.1% and 36.6%, respectively. Two states in these regions, Pará and Maranhão, showed an increase of 91.5% and 82.9% in the incidence of AIDS cases, respectively.^[10]^

Over the past 10 years, in fact, there has been an increase in new AIDS cases reported among men, especially those aged 15 to 19, 20 to 24, and 60 years of age and over. Focusing on an alarming increased rate of new cases among young people, from 2006 to 2015 the rate among 15 to 19 year olds more than tripled (2.4 to 6.7 cases/100,000 inhabitants) and among those from 20 to 24, doubled (15.9–33.1 cases/100,000). In the same period of 10 years, AIDS cases among MSM increased from 35.3% to 46.2% (31%) compared to all categories of AIDS cases reported among men.^[10]^

In 2009, the Brazilian Ministry of Health conducted the first National HIV Biological and Behavioral Surveillance Survey (BBSS) among MSM, female sex workers (FSWs), and drug users (DU) for HIV. The results showed HIV prevalence of 4.9% among DU,^[11]^ 5.8% among FSW,^[12]^ and 12.1% among MSM.^[13]^ Compared to HIV prevalence in the general population (estimated at 0.37%)^[2]^ HIV prevalence among DU was 13 times higher, among FSW was 16 times higher, and among MSM was 33 times higher. In 2016, the 2nd National BBSS was conducted with MSM as we report here. FSW and, for the first time, transgender women were included in this BBSS and reported in this journal.

The objective of this paper is to present the prevalence of HIV infection among MSM in the 12 cities in Brazil that participated in the BBSS in 2016, the main outcome of this survey.

## 2 Material and methods

**Figure 1:**
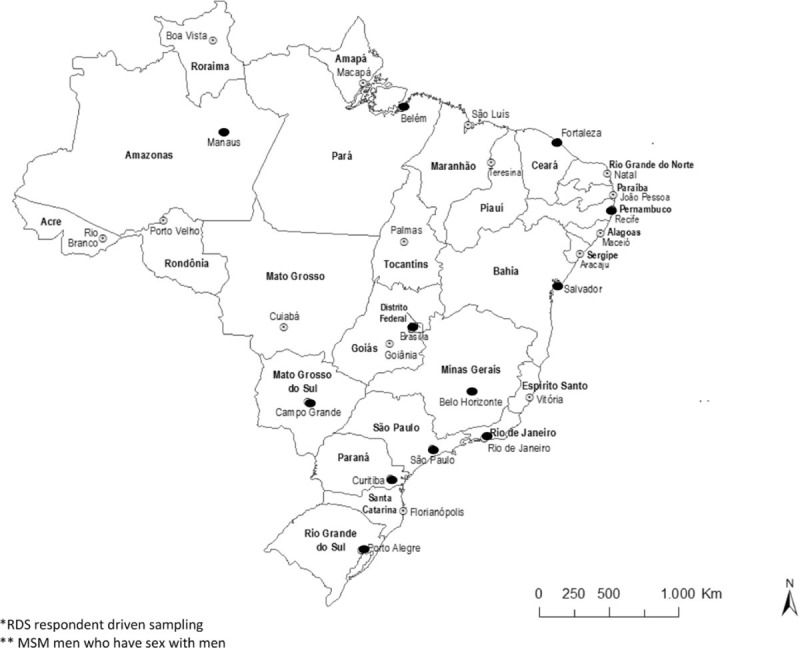
States and cities where RDS among MSM was conducted in 2016, Brazil. MSM = men who have sex with men, RDS = respondent-driven sampling.

The study used respondent-driven sampling (RDS) method to recruit participants and analyze results. RDS was chosen as the most appropriate method among available alternatives for reasons that included the large and mostly hidden social networks of MSM, and for comparison to Brazil's first HIV BBSS in 2006.^[14]^ Eligibility was limited to men 18 years of age or older reporting oral or anal sex with another man in the last 12 months, and residing, working, or studying in one of the 12 cities. The surveys were conducted from June to December 2016 in 12 Brazilian State capitals in the 5 Regions of Brazil. These were: Manaus, Belém, (North Region); Fortaleza, Recife, Salvador, (Northeast Region); Brasília, Campo Grande (Central West Region); Belo Horizonte, Rio de Janeiro, and São Paulo (Southeast Region); and Curitiba and Porto Alegre (South Region) (Fig. 1).

Formative research was conducted among 184 MSM between December 2015 and March 2016, to explore sex and gender identities, changes in HIV-related behaviors, organization of the MSM communities, and siting of the study office, incentive level, willingness to participate and provide a biological specimen, potential bottlenecks, and other operational issues. The BBSS was initiated with 5 to 6 seeds in each site selected to represent age and socioeconomic diversity within the MSM community. Only 2 sites added additional seeds when they felt recruitment was slowing (Table 1). Three coupons were distributed to each respondent to recruit others to the study. Each participant received a primary incentive of R$25 (25 Reais, the Brazilian currency or about US$7) and a secondary incentive of R$25 for each person recruited who completed the survey. Coupons and study IDs were managed with an on-line coupon generator developed as part of the data entry program. The social network question cascade is summarized in the following question: “How many men do you know, who also know you, who have had sex with other men (oral or anal) in the last 12 months, who live, study, or work in (municipality), are 18 years old or older, and that you encountered or spoke with in the last 2 months? Of these (repeat the number provided by the participant) how many would you invite to participate in this study?” The BBSS used Computer Assisted Personal Interview and all results were encrypted and uploaded to a password protected project database. Seeds were included in the sample for analysis.

**Table 1:**
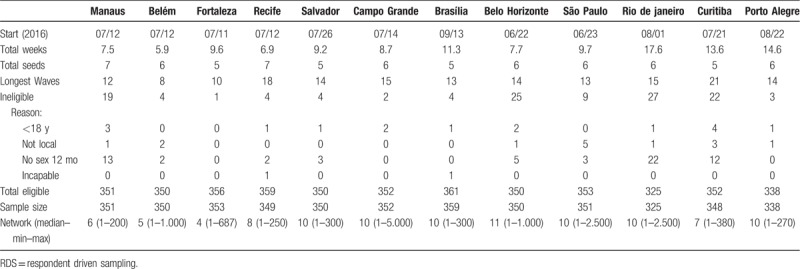
2016 RDS survey duration, seeds and longest wave, and eligibility by site (sample size n = 4176).

Following counseling, 2 tubes of venous blood were drawn. For HIV, blood was first tested with a rapid test for Anti-HIV antibodies (Alere/Bioeasy). If positive, the blood was tested with a second rapid test (Abon). Two positive results fulfilled Ministry criteria for reporting HIV positive serostatus. Respondents who tested positive for HIV were counseled and immediately referred to HIV/AIDS care centers in each of the 12 cities.

## 3 Analysis

HIV prevalence was the result of the constructed variable of the 2 positive rapid HIV tests, the criterion used by the Ministry of Health. An additional HIV prevalence was estimated by adding individuals who refused to test, reported positive HIV status, and who were taking antiretrovirals. Gile successive sampling estimator^[15]^ was used to produce weighted estimates of both prevalence rates using RDS analyst.^[16]^ To calculate a single nation-wide HIV prevalence, data from the 12 cities were merged and analyzed with Stata 14.0 complex survey data analysis tools in which each city was treated as its own strata.

## 4 Ethical considerations

The overall study was approved by the Committee on Research Ethics of the Federal University of Ceará, accredited by the National Commission on Research (#1.024.053(23/06/2015)). All respondents signed a consent form to participate in the interview and separately consented for each test that was offered.

## 5 Results

Data were collected from June 23, 2016 in Belo Horizonte to December 2, 2016, in Rio de Janeiro and Porto Alegre (Table 1). Average time to completion was 10.2 weeks, with Belém finishing in 6 weeks and Rio de Janeiro requiring 17.6 weeks. Median network size of members who might be recruited varied from 4 to 10. Longest chain length varied from 8 waves in Belém to 21 waves in Curitiba. There were relatively few ineligible participants, ranging from 1 in Fortaleza to 27 in Rio de Janeiro. Rio de Janeiro and Porto Alegre did not achieve the sample size of 350 designated by the Ministry of Health.

Our sample was young (58.3% < 25; 95%CI: 54.6–62.0), a majority of mixed ethnicity (42.0%; 95%CI: 38.5–45.6), and relatively well educated, with 59.3% having completed high school (95%CI: 55.7–62.8) (Table 2). Using the standard socioeconomic strata (A–E) developed by the Brazilian Association of Research Organizations,^[17]^ our sample was majority middle (C) (43.0%; 95%CI: 39.4–46.7) and lower (D, E) strata (16.2%; 95% CI: 13.8–19.0). Single men constituted 83.0% of the sample (95% CI: 80.1–85.6).

**Table 2:**
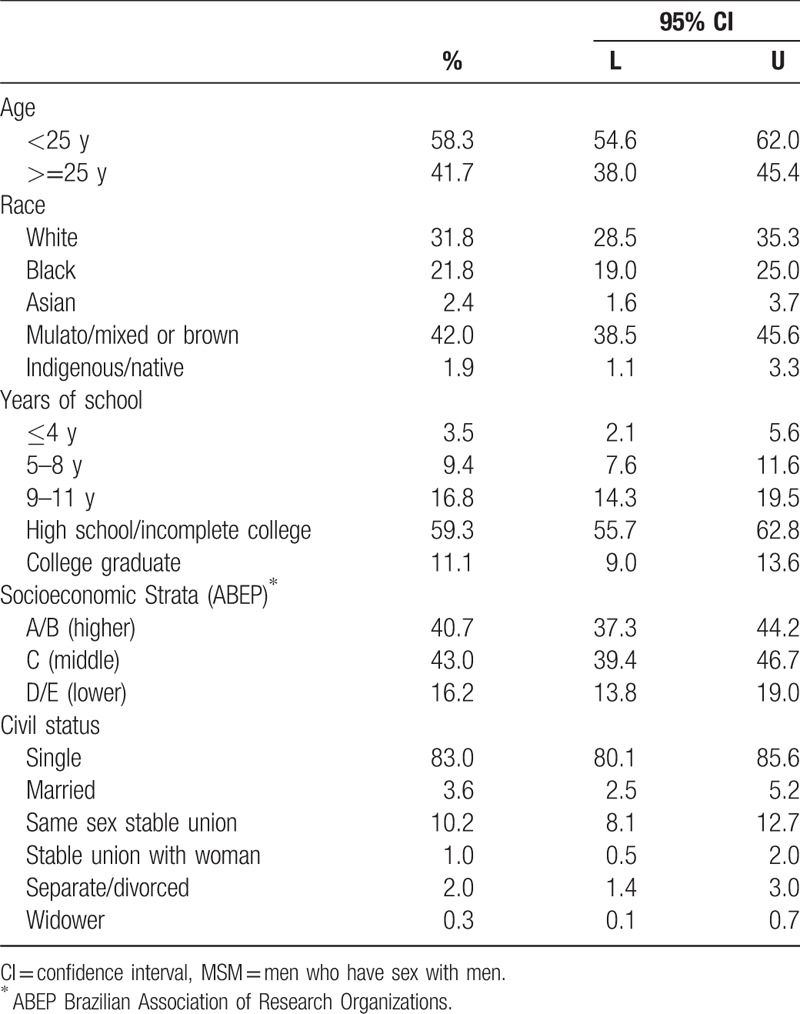
Summary of socioeconomic and demographic characteristics of MSM participants in 12 cities, Brazil 2016.

A total of 3958 of the 4176 respondents agreed to test for HIV (90.2%) (95%CI: 87.3–92.4) (Table 3). For results without imputation as described above, 17.5% (95%CI: 14.7–20.7) of our sample were HIV positive. With imputation, 18.4% (95%CI: 15.4–21.7) were seropositive (Table 4). There was important variation among cities, with Brasília reporting the lowest prevalence (5.8%; 95%CI: 3.5–9.6) and São Paulo the highest (24.8%; 95%CI: 18.5–32.4), maintaining these positions with and without imputation (Table 4).

**Table 3:**
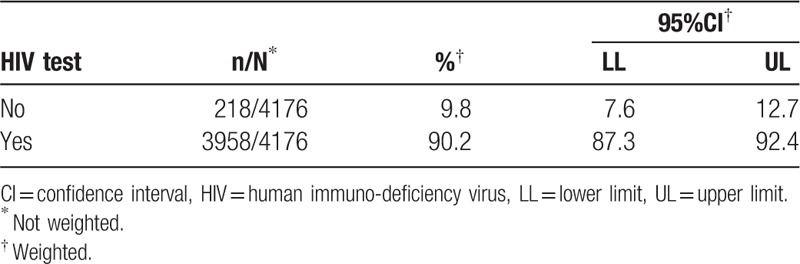
Tested for HIV in the study.

**Table 4:**
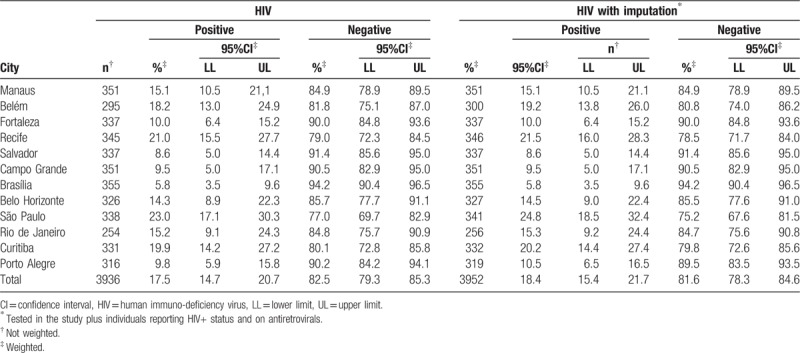
HIV test in MSM with and without imputation by city.

## 6 Discussion

This report joins Brazil to a growing number of countries demonstrating high levels of HIV prevalence among MSM.^[3,18]^ These reports challenge the initial optimism for test and treat strategies for controlling and ultimately eliminating HIV.^[19–22]^ Although successful treatment will increase prevalence there is evidence of increasing incidence and important gaps in prevention, including a fall-off in the promotion and use of condoms and other preventive behaviors.^[1]^ Available data on HIV prevalence and incidence from low, middle, and high-income countries suggest that the HIV epidemics among MSM are increasing due to stigma and discrimination, sexual behavior, and issues with adherence and care-seeking.^[23]^ From its onset, the HIV epidemic in Brazil has been a concentrated epidemic, with stable prevalence rates around 0.37%^2^ for the general population, and above 5% prevalence rates among MSM, FSWs, and illicit DU. Structural barriers, conservative social and religious movements in government and insufficient allocation of funds from local governments, may have contributed to the deficits in primary prevention programs reported in Brazil.^[1]^

Our study shows higher levels of HIV prevalence among the MSM in the 12 cities (18.4%; CI95%: 15.4–21.7) we studied compared to the BBSS we conducted in 2009 in 10 cities (12.1%; 95%CI: 10.0–14.5) suggesting a potential increase in HIV incidence. In our discussion, we explore several potential reasons for rising seroprevalence.

Accounting for reasons for nation-wide changes in HIV prevalence across time is not a simple task.^[24]^ The causes of changes in the HIV epidemic are inherently multidimensional, involving environments of vulnerability and risk, stigma and discrimination for key populations, and changing behaviors, policies, and programs. During the period between our 2 surveys there have been major new strategies to address HIV/AIDS programs and important changes in the institutional, social, and political context in Brazil. Many of these changes – positive and negative – are well documented in Malta and Beyrer.^[25]^

In addition to changes in these contexts, the changes in sexual behavior among the youth in our sample, shown in accompanying paper in this journal, is also concerning. In addition, the formative research for our study uncovered a catchphrase used by youth: “AIDS já não me assusta mais” (AIDS does not scare me anymore).^[26]^ Understanding this comment requires understanding the new scenario for AIDS created by antiretrovirals and the “treatment to prevention” initiatives^[27,28]^ such as early treatment and preexposure prophylaxis. These initiatives have resulted in a medicalized approach that treats HIV infection as a lifelong chronic condition.^[29]^ In parallel, NGOs that addressed MSM and AIDS prevention have been defunded, removing spaces for community organizing around prevention and testing, and for generating solidarity among the MSM communities.^[30,31]^ Growing support in the Brazilian government for the “Bullets, Beef, and Bible” caucus, in the most conservative congress in the Brazil democratic era, has led to a regressive gender and sexuality agenda and reduced support for programs that focus on MSM needs.^[25,30,32]^ In fact, there have been major budget cuts or dismantling of programs in research, prevention, and treatment throughout the health sector.^[33]^

Our 2016 sample is younger than the 2009 sample.^[13]^ Although HIV prevalence increases with increasing age due to cumulative incidence and improved survival, the much higher prevalence in 2016 is particularly notable. The trend toward rising new infections among youth is not isolated to Brazil. The US Centers for Disease Control report that in the US youth aged 13 to 24 account for 20% of all new HIV diagnoses, 81% of those occurring among self-reporting gay and bisexual males. Youth presents a special problem, reports CDC: they are the least likely to test or to use a condom, are more likely to drink or use drugs during sex, and have 4 or more partners during their incipient sexual careers.^[34]^ Other studies confirm these differences between younger and older MSM. Analyzing from a generational perspective, Méthy et al^[35]^ report for France that the younger generation of MSM are more likely to have their first sex with a man compared to the older generation of MSM, who were youth in the 1980s. Reports of oral and anal sex, and frequency of sex were lower for older generations of MSM, but are higher among the younger generation today. Interestingly, Wall et al note for the US similarities in this increased sexual frequency in both young MSM and heterosexuals.^[36]^

### 6.1 Limitations

This is a repeated sample among MSM in Brazil using RDS. Limitations of RDS have been well documented.^[15,37,38]^ A recent publication has also criticized the reliability of RDS when repeated in the same population in a relatively short period of time.^[39]^ Because the sample selected through RDS is a product of a series of both theoretical assumptions and operational issues, such as seed selection, logistics, and control of “masking,” two consecutive samples could differ substantially. Khatib et al^[39]^ in discussing reproducibility refer to an earlier study in Zanzibar.^[40]^ In the latter study non-MSM IDUs masking as MSM entered the study and drove up the seroprevalence rate. For this reason, formative research and monitoring,^[41]^ applying a large design effect,^[42]^ adherence to STrengthening the Reporting of OBservational studies in Epidemiology (STROBE) RDS guidelines,^[43]^ and RDS diagnostics are proposed.^[37]^ Until better methods are available, RDS will continue to be used to provide population estimates of hard to reach populations in the HIV epidemic. Even though our sample is different from the one in 2009, our findings move in the same direction as other sources of information.^[2,44,45]^

## 7 Conclusions

We take the opportunity in this paper to not just focus on our numbers, but advocate for a response. Our findings present a serious challenge to policy makers: how are we to address the increasing epidemic among MSM in Brazil? Our results argue for an invigorated prevention effort combining innovative approaches such as engaging communities in developing solutions and involving communities themselves in research, publication, and enhanced advocacy.^[46]^ Such strategies are part of a new sustainable development agenda, together with investment in science, innovative solutions, national and local leadership, and strong political commitment to achieve these targets. Parker terms this approach “prevention literacy” to complement treatment literacy, building on the strategy that used NGOs and community participation to promote prevention.^[47]^ Preventing AIDS makes sense on so many levels, but for governments actively shrinking health budgets, reducing transmission makes ultimate sense.

## Acknowledgments

The authors thank the financial support provided by the Brazilian Ministry of Health, through the Secretariat for Health Surveillance and the Department of Prevention, Surveillance and Control of Sexually Transmitted Infections, HIV/AIDS and Viral Hepatitis. The authors also thank all the respondents and their enthusiastic participation in this troubled time for sexual minorities, without them this study would not be possible.

## Author contributions

**Conceptualization:** Ligia Kerr, Carl Kendall, Mark Drew Crosland Guimarães, Adele Schwartz Benzaken, Cristina Pimenta, Ana Roberta Pati Pascom, Willi Mcfarland, Lisa Grazina Johnston.

**Data curation:** Ligia Kerr, Rosa Maria Salani Mota.

**Formal analysis:** Ligia Kerr, Mark Drew Crosland Guimarães, Rosa Maria Salani Mota.

**Funding acquisition:** Ligia Kerr.

**Investigation:** Ligia Kerr, Carl Kendall, Mark Drew Crosland Guimarães, Maria Amelia Veras, Ines Dourado, Ana Maria de Brito, Edgar Merchan-Hamann, Alexandre Kerr Pontes, Andréa Fachel Leal, Daniela Riva Knauth, Ana Rita Coimbra Motta-Castro, Raimunda Hermelinda Maia Macena, Luana Nepomuceno Costa Lima, Lisangela Cristina Oliveira, Ximena Pamela Diaz Bermudez, Regina Célia Moreira, Luiz Fernando Macedo Brígido.

**Methodology:** Ligia Kerr, Carl Kendall, Mark Drew Crosland Guimarães, Rosa Maria Salani Mota, Regina Célia Moreira.

**Project administration:** Ligia Kerr, Mark Drew Crosland Guimarães, Maria Amelia Veras, Ines Dourado, Ana Maria de Brito, Edgar Merchan-Hamann, Alexandre Kerr Pontes, Daniela Riva Knauth, Ana Rita Coimbra Motta-Castro, Raimunda Hermelinda Maia Macena, Luana Nepomuceno Costa Lima, Lisangela Cristina Oliveira, Ana Claudia Camilo.

**Supervision:** Ligia Kerr, Mark Drew Crosland Guimarães, Maria Amelia Veras, Ines Dourado, Ana Maria de Brito, Edgar Merchan-Hamann, Alexandre Kerr Pontes, Andréa Fachel Leal, Daniela Riva Knauth, Ana Rita Coimbra Motta-Castro, Raimunda Hermelinda Maia Macena, Luana Nepomuceno Costa Lima, Lisangela Cristina Oliveira, Regina Célia Moreira, Ana Claudia Camilo.

**Visualization:** Ligia Kerr.

**Writing – original draft:** Ligia Kerr, Carl Kendall, Luana Nepomuceno Costa Lima.

**Writing – review & editing:** Ligia Kerr, Carl Kendall, Mark Drew Crosland Guimarães, Rosa Maria Salani Mota, Maria Amelia Veras, Ines Dourado, Ana Maria de Brito, Edgar Merchan-Hamann, Alexandre Kerr Pontes, Andréa Fachel Leal, Daniela Riva Knauth, Ana Rita Coimbra Motta-Castro, Raimunda Hermelinda Maia Macena, Lisangela Cristina Oliveira, Adele Schwartz Benzaken, Gerson Pereira, Cristina Pimenta, Ana Roberta Pati Pascom, Ximena Pamela Diaz Bermudez, Regina Célia Moreira, Luiz Fernando Macedo Brígido, Ana Claudia Camilo, Willi Mcfarland, Lisa Grazina Johnston.
